# Supplementary material for: ACE2-like carboxypeptidase B38-CAP protects from SARS-CoV-2-induced lung injury
Source: Nat Commun. 2021 Nov 23;12:6791. doi: 10.1038/s41467-021-27097-8 (PMC8610983; doi:10.1038/s41467-021-27097-8)
Supplement: Supplementary file 1 — Supplementary information [file 41467_2021_27097_MOESM1_ESM.pdf]

Tomokazu Yamaguchi, et al. "ACE2-like carboxypeptidase B38-CAP protects from SARS-CoV-2-induced lung injury"

Supplementary Information:

Supplementary Figure 1-8.

Supplementary Table 1-5.

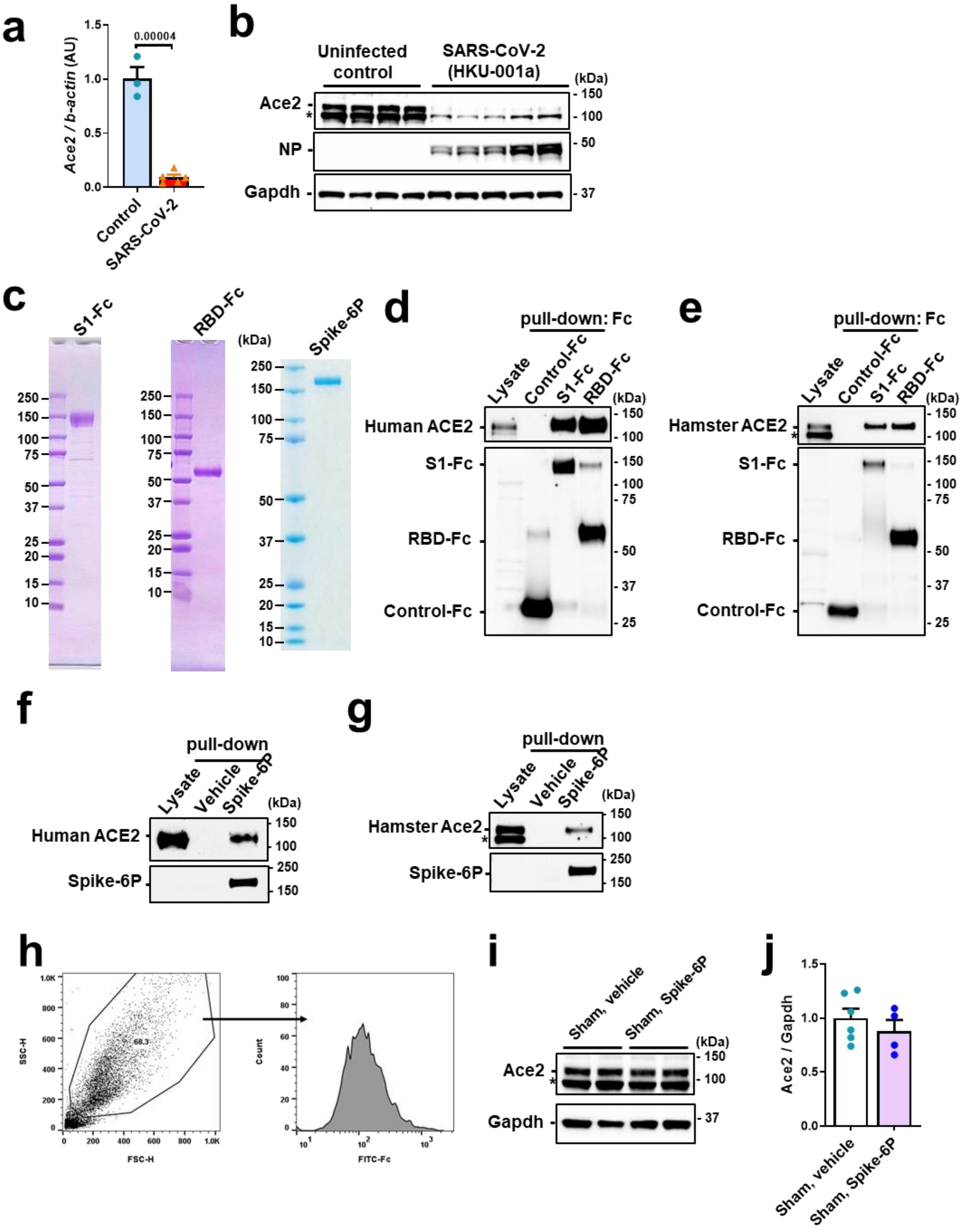

Supplementary Figure 1. Downregulation of ACE2 expression by SARS-CoV-2 (HKU-001a) and recombinant Spike proteins

a, mRNA expression of Ace2 in hamster lungs at 4 days after intranasal infection of SARS-CoV-2 (UT-NCGM02). Uninfected control hamsters (n = 3) and SARS-CoV-2 infected hamsters (n = 5).

Two-tailed unpaired *t*-test. **b**, Protein expression of Ace2 in hamster lungs at 4 days after intranasal infection of SARS-CoV-2 (Hong Kong strain, HKU-001a). Representative Western Blot is shown. **c,** Preparation of recombinant Spike proteins. SDS-PAGE of purified Spike proteins; S1-Fc, RBD-Fc and Spike-6P are shown. **d-g**, Binding of recombinant S1-Fc or RBD-Fc protein to human ACE2 and hamster Ace2 in pull-down assays. Western Blot of human ACE2 (**d, f**, *top*), hamster Ace2 (**e, g**, *top*) and respective Fc proteins (**d, e**, *bottom*) or Spike-6P (**f, g**, *bottom*) are shown. Total lysates are shown as controls. *; unfolded or short form of ACE2 or non-specific band. **h**, An example of gating strategy to determine the percentage of Vero E6 cells bound by RBD-Fc or control-Fc in Figure 2c,d. FSC/SSC plot was gated for live and healthy Vero E6 cells. **i-j**, ACE2 protein expression in hamster lungs. Representative Western blot (**i**) and quantification of ACE2 protein abundance (**j**) are shown. (n = 6 hamsters for Sham + vehicle and n = 4 for Sham + Spike-6P). *; unfolded form of ACE2 or non-specific band. All values are means ± SEM. Two-tailed unpaired *t*-test. Independent experiments were performed one time (**a-h**) or two times (**i-j**), and consistent results were obtained.

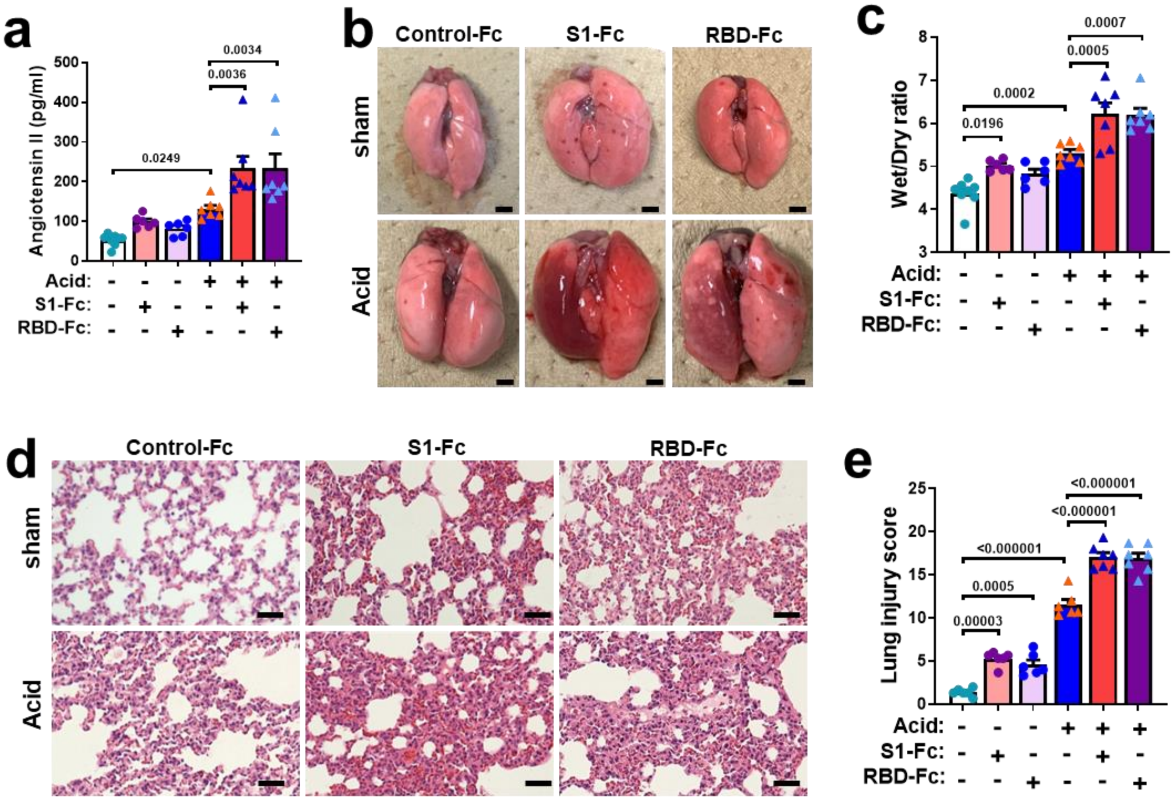

Supplementary Figure 2. The SARS-CoV-2 Spike proteins worsen acid-induced lung injury. **a,** Measurements of Ang II in the plasma of hamster with ELISA (n = 8 hamsters for sham + control-Fc, n = 6 each for sham + S1-Fc or sham + RBD-Fc, n = 7 each for other experimental groups). **b**, Representative photograph of the lungs of hamsters under acid and Spike protein (S1-Fc or RBD-Fc)-induced lung injury in the experimental protocol (Fig. 1g). Bars indicate 2 mm. **c**, Wet to dry weight ratios of lungs as readout for pulmonary edema in control, S1-Fc or RBD-Fc–treated hamsters in the presence or absence of acid-induced lung injury (n = 8 hamsters for sham + control-Fc, n = 6 each for sham + S1-Fc and sham + RBD-Fc, n = 7 each for other experimental groups). **d**, Lung histopathology. Representative images are shown. Bars indicate 100 μm. **e,** Lung injury score (n = 7 hamsters each for Acid + S1-Fc or Acid + RBD-Fc, n = 6 each for other experimental groups). All values are means ± SEM. Two-way ANOVA with Sidak's multiple comparisons test. Numbers above square brackets show significant *P* values. Independent experiments were performed three times (**a-e**), and consistent results were obtained.

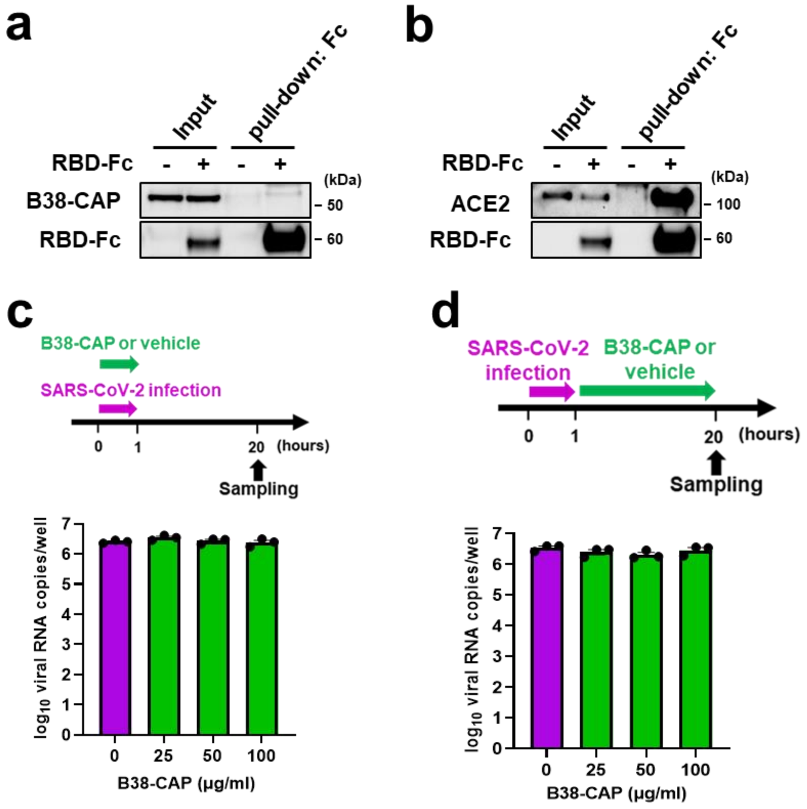

Supplementary Figure 3. B38-CAP neither binds to Spike RBD nor neutralize cell entry of SARS-CoV-2 in vitro.

a-b, *In vitro* binding of recombinant RBD-Fc protein to recombinant soluble human ACE2 protein but not B38-CAP in pull-down assays. Western Blot of B38-CAP (**a**) and soluble human ACE2 (**b**) are shown. **c-d,** No effects of B38-CAP on SARS-CoV-2 replication in Vero E6/TMPRSS2 cells in vitro. Different concentrations of B38-CAP were added with SARS-CoV-2 (MOI 0.05) to the culture medium of Vero E6/TMPRSS2 cells for 1 hour and then cells were washed and incubated with fresh medium (**c**). Effect of B38-CAP treatment on progeny virus was examined by adding B38-CAP the culture of Vero E6/TMPRSS2 cells after SARS-CoV-2 infection (MOI 0.05) for 1 hour and subsequent wash out (**d**). Cell lysates were harvested at 20 hours post infection, and viral RNA was assayed by qRT-PCR. All values are means ± SEM. One-way ANOVA with Sidak's multiple comparisons test. Independent experiments were performed one time (**c, d**) or two times (**a, b**), and consistent results were obtained.

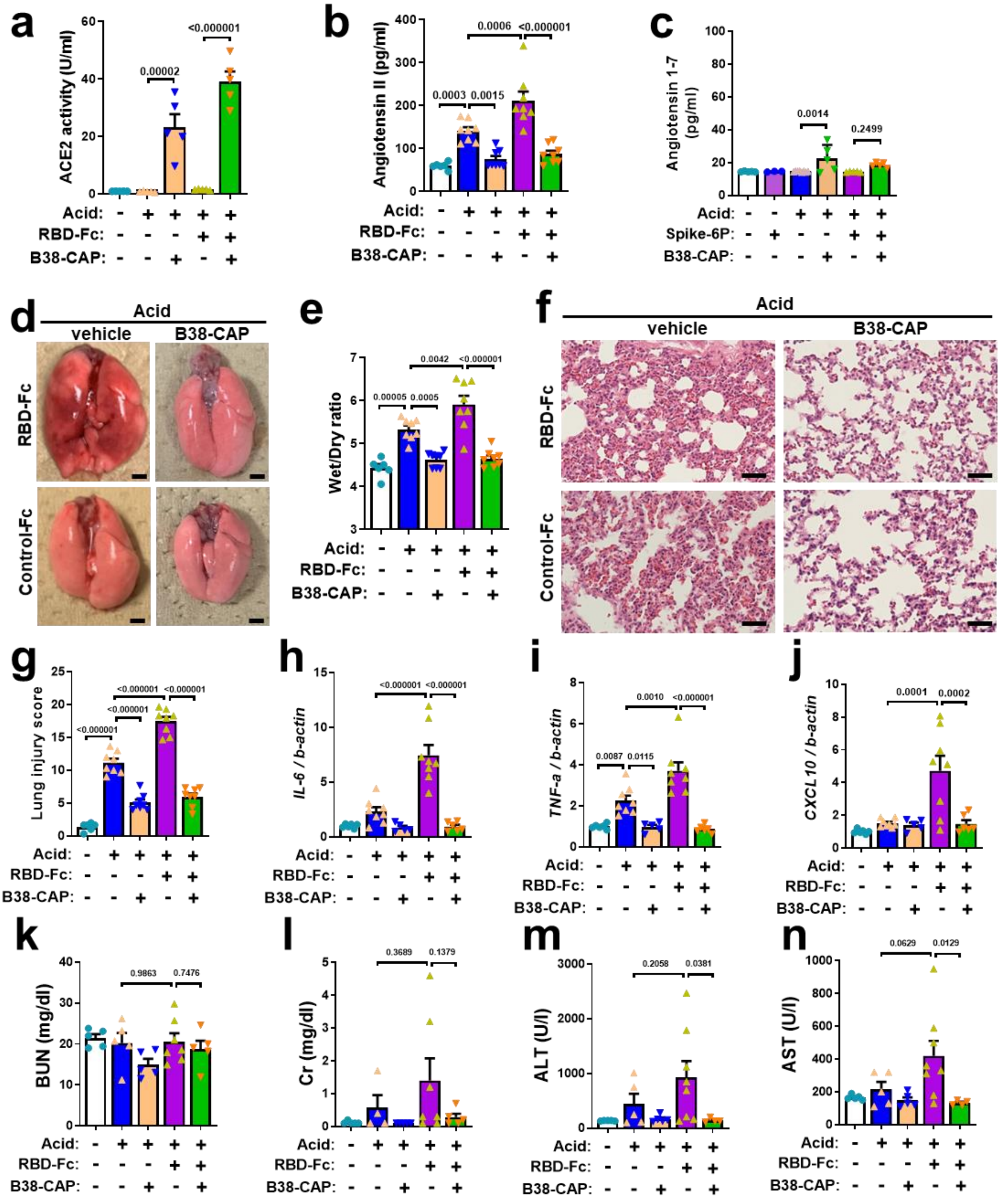

Supplementary Figure 4. B38-CAP mitigates SARS-CoV-2 Spike protein-induced lung injury. **a**, Measurements of ACE2 activity in the plasma of hamsters at 17 hours after acid instillation in the experimental protocol shown in Fig. 2a (n = 5 per group). The enzymatic activity was measured

with the fluorogenic ACE2 substrate, Nma-His-Pro-Lys(Dnp), and non-specific activity in the assay was excluded by subtracting the values measured with the ACE2 inhibitor MLN-4760 from the values without MLN-4760. **b,** Plasma Ang II levels at 24 hours after acid instillation (n = 6 hamsters for sham + control-Fc + vehicle, n = 8 each for other experimental groups). **c,** Plasma Ang 1-7 levels at 24 hours after acid instillation (n = 5 hamsters for sham + vehicle, n = 3 for sham + Spike-6P and n = 8 each for other experimental groups). **d,** Representative photograph of hamster lungs. Bars indicate 2 mm. **e**, Wet to dry weight ratios of lungs at 24 hours after acid instillation (n = 6 hamsters for sham + control-Fc + vehicle and n = 8 each for other experimental groups). **f-g**, Lung histopathology. Tissue samples were harvested at 24 hours after acid instillation. Representative images are shown (**f**). Bars indicate 100 μm. Lung injury score measurements (**g**) (n = 6 hamsters for sham + control-Fc + vehicle and n = 8 each for other experimental groups). **h-j,** qRT-PCR analysis of pro-inflammatory cytokine expression in the lungs of hamsters; mRNA levels of *IL-6* (**h**), *TNF-α* (**i**) and *CXCL10* (**j**) normalized with *β-actin* (n = 6 hamsters each for sham + control-Fc + vehicle or Acid + RBD-Fc + B38-CAP, n = 8 each for Acid + control-Fc + vehicle or Acid + RBD-Fc + vehicle and n = 5 for Acid + control-Fc + B38-CAP). **k-l**, Kidney function assessment with measurements of BUN (**k**) and Creatinine (Cr) (**l**) in the blood. n = 7 hamsters for Acid + RBD-Fc + vehicle and n = 5 each for other experimental groups (**k**). n = 4 hamsters for Acid + control-Fc + vehicle, n = 7 for Acid + RBD-Fc + vehicle and n = 5 each for other experimental groups (**l**). **m-n**, Liver function test with measurements of alanine aminotransferase (ALT) (**m**) and aspartate aminotransferase (AST) (**n**) in the blood. n = 8 hamsters for Acid + RBD-Fc + vehicle, n = 4 for Acid + RBD-Fc + B38-CAP and n = 5 each for other experimental groups. All values are means ± SEM. One-way ANOVA with Sidak's multiple comparisons test. Numbers above square brackets show significant *P* values. Independent experiments were performed three times (**a-n**), and consistent results were obtained.

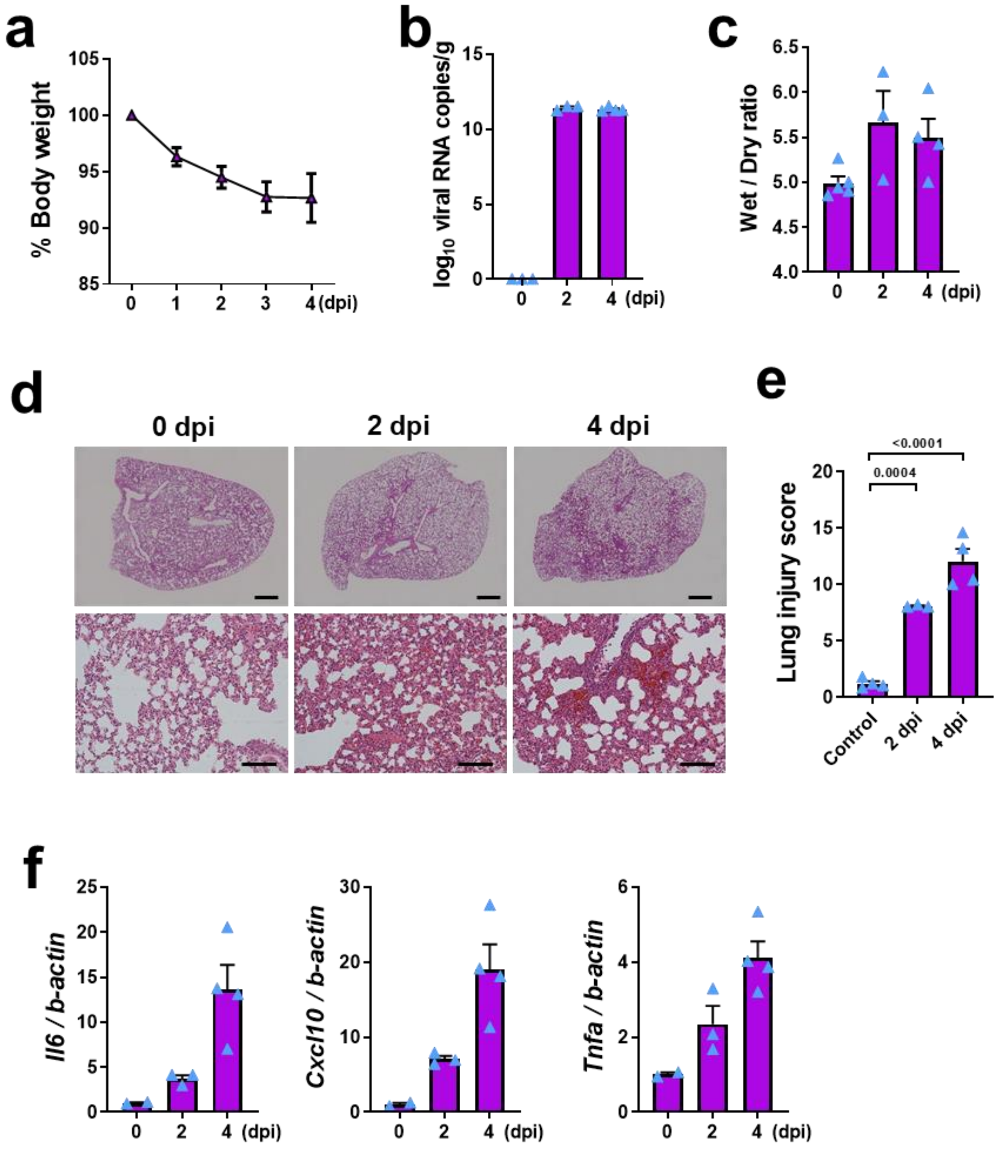

Supplementary Figure 5. SARS-CoV-2-induced lung injury in hamsters.

a, %Changes of body weight of hamsters after intratracheal infection of SARS-CoV-2 (1 x 10^3 $TCID_{50}$) at 0 dpi (n = 3), 2 dpi (n = 3) and 4 dpi (n = 4). dpi; days post infection. **b**, qRT-PCR of virus N gene expression in the lungs of hamsters at 0 dpi (n = 5), 2 dpi (n = 3) and 4 dpi (n = 4). **c**, Lung weight to body weight ratio. **d-e**, Lung histopathology. Representative images are shown (**d**). Bars indicate 1 mm (*upper*) and 100 μm (*bottom*). Lung injury scores were measured at 0 dpi (n = 4), 2 dpi (n = 3) and 4 dpi (n = 4) (**e**). **f,** qRT-PCR analysis of pro-inflammatory cytokine expression

in the lungs of hamsters; mRNA levels of IL-6 (*Il6*), CXCL10 (*Cxcl10*) and TNF-α (*Tnfa*) normalized with β-actin (*b-actin*) at 0 dpi (n = 2), 2 dpi (n = 3) and 4 dpi (n = 4). All values are means ± SEM. One-way ANOVA with Sidak's multiple comparisons test. Numbers above square brackets show *P* values. Independent experiments were performed two times (**a-f**), and consistent results were obtained.

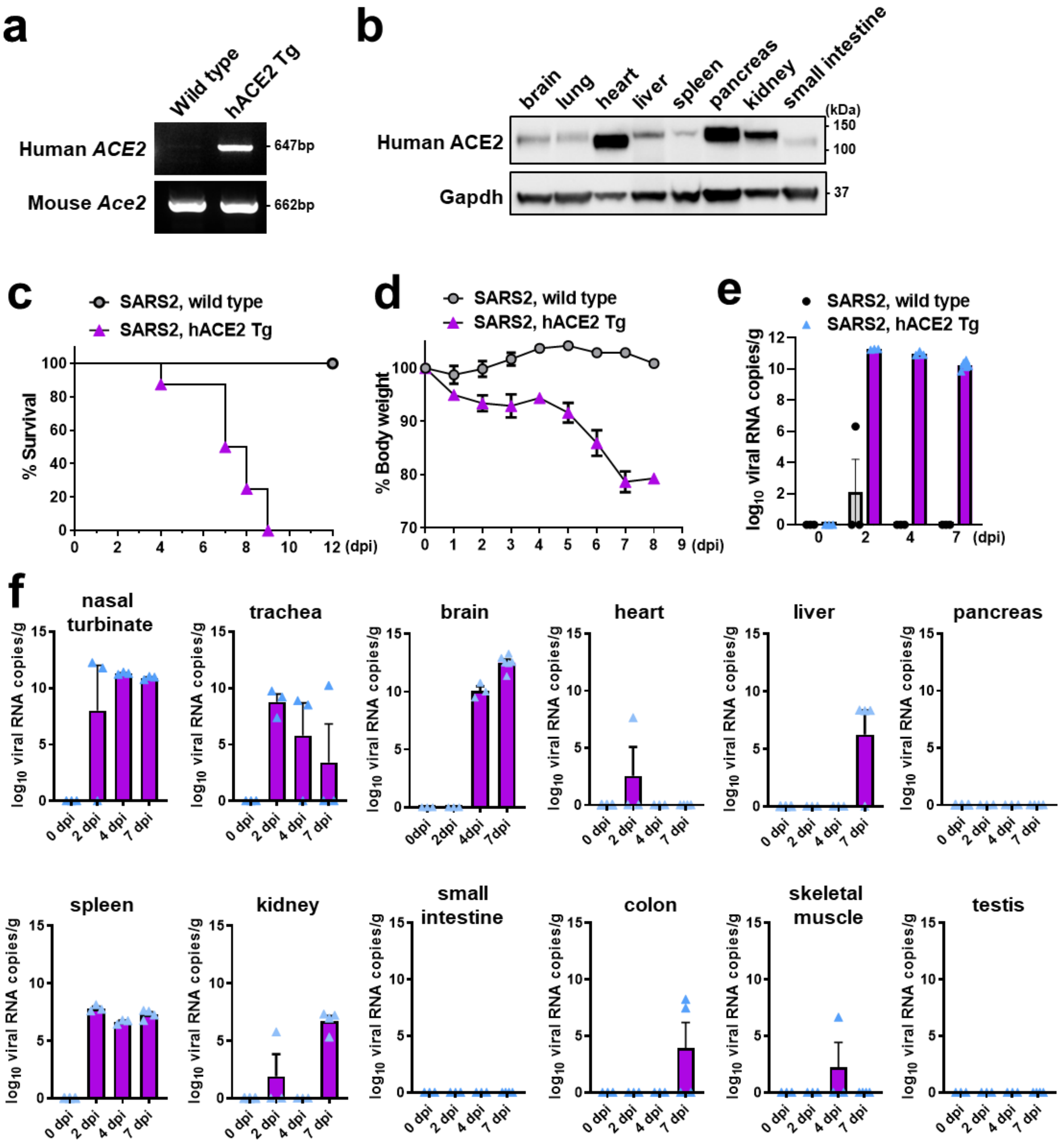

Supplementary Figure 6. SARS-CoV-2 infection in hACE2 Tg mice.

a, Genotyping PCR of hACE2 Tg mice. Human ACE2 transgene was detected in the genomic DNA from tail of hACE2 Tg mouse but not in that of wild type mouse. Endogenous mouse Ace2 gene served as a control. **b**, Protein expression of human ACE2 in the tissues of uninfected hACE2 Tg mice. Western blot was done with anti-human ACE2 antibody, which does not cross-react with

mouse Ace2 as shown in Fig. 4a. **c-d**, Intratracheal infection of SARS-CoV-2 (2 x 10^3 $TCID_{50}$) in hACE2 Tg mice (n = 8) and wild type mice (n = 5). %Survival of the mice (**c**) and %changes of body weight (**d**) after infection of SARS-CoV-2 are shown. **e-f**, Kinetics of viral load in the lungs (**e**) and other organs (**f**) of infected hACE2 Tg mice at 0 dpi (n = 3), 2 dpi (n = 3), 4 dpi (n = 4) and 7 dpi (n =4) and wild type mice (n = 3 for each time points). Viral N RNA copy number of were measured. All values are means ± SEM. Independent experiments were performed one time (**b, f**) or two times (**a**, **c-e**), and consistent results were obtained.

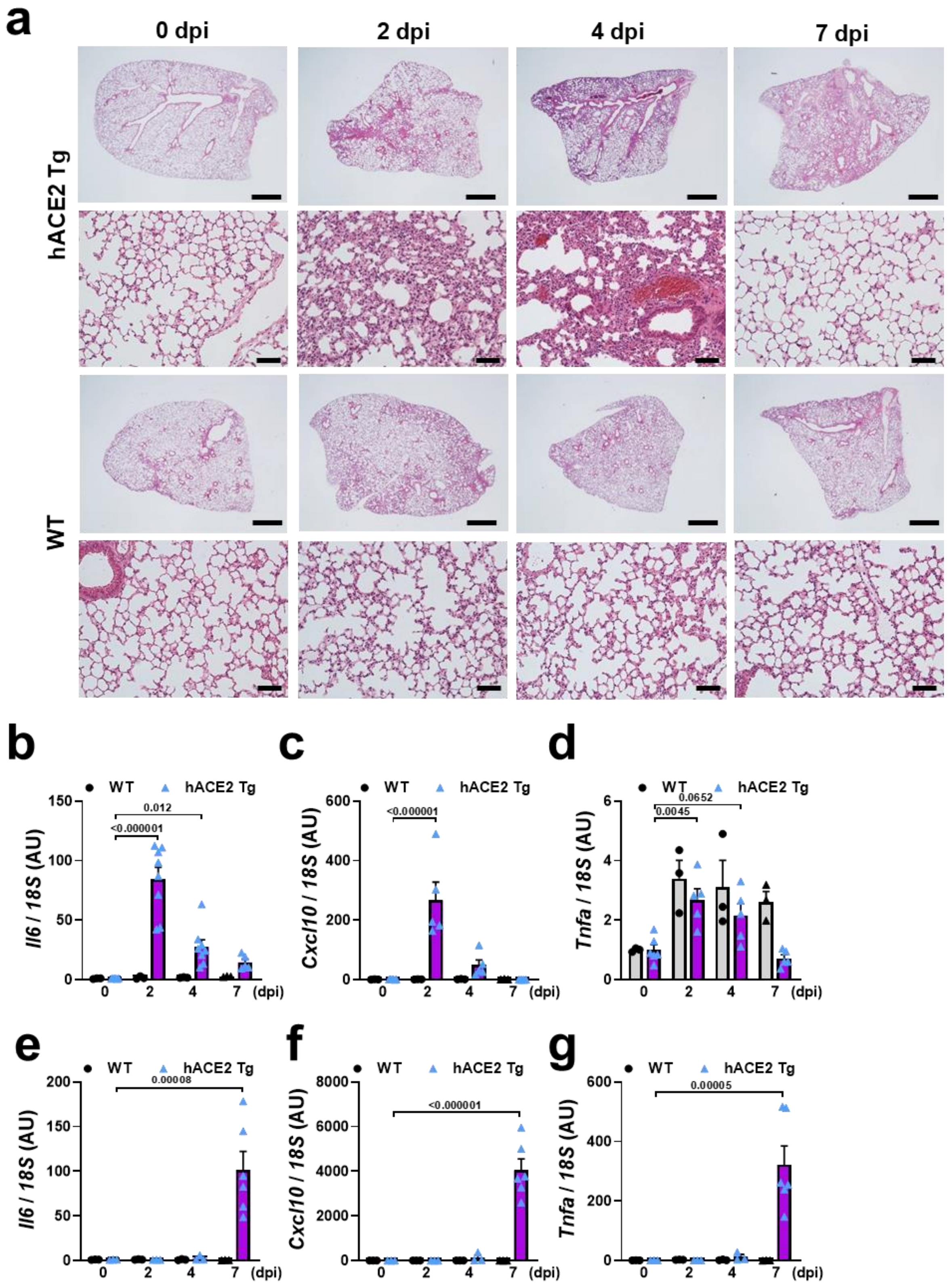

Supplementary Figure 7. SARS-CoV-2 induces lung injury in hACE2 Tg mice.

a, Lung histopathology of SARS-CoV-2 infected hACE2 Tg mice or wild type mice. Representative images are shown (**a**). Bars indicate 1 mm (*upper*) and 100 μm (*bottom*). **b-g,** qRT-PCR analysis of pro-inflammatory cytokine expression in the lungs (**b-d**) and brains (**e-g**) of SARS-CoV-2 infected hACE2 Tg mice; mRNA levels of IL-6 (*Il6*) (**b, e**), CXCL10 (*Cxcl10*) (**c, f**) and TNF-α (*Tnfa*) (**d, g**) normalized with 18S. n = 6 for hACE2 Tg mice at 0 dpi, n = 8 each for hACE2 Tg mice at 2 dpi and 4 dpi, n = 7 for hACE2 Tg mice at 7 dpi and n = 3 for wild type mice at each time points (**b**). n = 6 for hACE2 Tg mice at 0 dpi, n = 5 each for hACE2 Tg mice at 2 dpi, 4 dpi and 7 dpi and n = 3 for wild type mice at each time points (**c, d**). n = 6 for hACE2 Tg mice at 7 dpi, n = 3 each for hACE2 Tg mice at 0 dpi, 2 dpi, and 4 dpi or wild type mice at each time points (**e-g**). All values are means ± SEM. One-way ANOVA with Sidak's multiple comparisons test. Numbers above square brackets show *P* values. Independent experiments were performed two times (**a-g**), and consistent results were obtained.

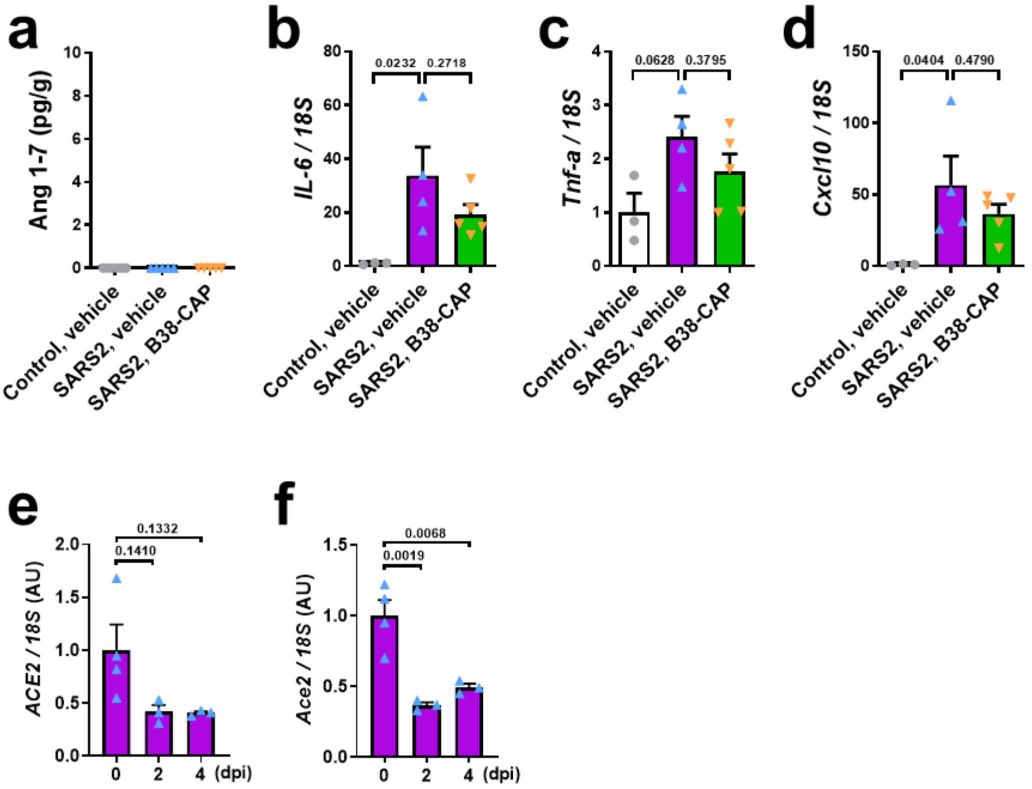

Supplementary Figure 8. SARS-CoV-2 induces lung injury in hACE2 Tg mice.

a, Angiotensin 1-7 levels in the lung tissues (n = 6 mice for control + vehicle, n = 5 for SARS2 + vehicle and n = 5 for SARS2 + B38-CAP). **b-d,** qRT-PCR analysis of pro-inflammatory cytokine expression in the lungs of SARS-CoV-2 infected hACE2 Tg mice; mRNA levels of IL-6 (*IL-6*), CXCL10 (*Cxcl10*) and TNF-α (*Tnf-a*) normalized with 18S (n = 3 mice for control + vehicle, n = 4 for SARS2 + vehicle and n = 5 for SARS2 + B38-CAP). **e-f,** mRNA expression of human ACE2 (**e**) and mouse Ace2 (**f**) were normalized with 18S (n = 4 mice for 2 dpi, n = 3 each for 2 and 4 dpi). All values are means ± SEM. One-way ANOVA with Sidak's multiple comparisons test. Numbers above square brackets show *P* values. Independent experiments were performed two times (**a-f**), and consistent results were obtained.

Supplementary Table 1.
Primer list

qRT-PCR primers

species	genes	5'-Sense-3'	5'-Antisense-3'	5'-Probe-3'
Hamster	*IL-6*	GGACAATGACTATGTGTTGTTAGAA	AGGCAAATTTCCCAATTGTATCCAG	
Hamster	*TNF-a*	TGAGCCATCGTGCCAATG	AGCCCGTCTGCTGGTATCAC	
Hamster	*CXCL10*	CTCTACTAAGAGCTGGTCC	CTAACACACTTTAAGGTGGG	
Hamster	*b-actin*	TGTACCCAGGCATTGCTGAC	TCATCGTACTCCTGCTTGCTGA	
Mouse	*IL-6*	CAACGATGATGCACTTGCAGA	CTCCAGGTAGCTATGGTACTCCAGA	
Mouse	*TNF-a*	GCCTCTTCTCATTCCTGCTTG	CTGATGAGAGGGAGGCCATT	
Mouse	*Cxcl10*	GCCGTCATTTTCTGCCTCAT	GCTTCCCTATGGCCCTCATT	
Mouse	*18S*	AAACGGCTACCACATCCAAG	CCTCCAATGGATCCTCGTTA	
SARS-CoV-2	*N*	AAATTTTGGGGACCAGGAAC	TGGCAGCTGTGTAGGTCAAC	FAM-ATGTCGCGCATTGGCATGGA-BHQ

Genotyping PCR primers

strain	Genes	5'-Sense-3'	5'-Antisense-3'
hACE2 Tg	*human ACE2*	CTTGGTGATATGTGGGGTAGA	CGCTTCATCTCCCACCACTT
Mouse	*mouse Ace2*	CCGGCTGCTCTTTGAGAGGACA	CTTCATTGGCTCCGTTTCTTAGC

Supplementary Table 2.
Lung injury score for hamster with Spike-6P plus acid-induced lung injury

Group	Alveolar congestion	Hemorrhage	Neutrophil infiltrates	Alveolar wall thickness	Hyaline Membrane formation	Total scores
Control, vehicle	0.00 ± 0.00	0.47 ± 0.13	0.87 ± 0.08	0.73 ± 0.07	0.00 ± 0.00	2.07 ± 0.24
Control, Spike-6P	1.33 ± 0.51	1.78 ± 0.59	1.11 ± 0.22	1.11 ± 0.11	0.33 ± 0.00	5.67 ± 1.35
Acid, vehicle	1.75 ± 0.22	1.67 ± 0.21	2.71 ± 0.16	2.50 ± 0.23	1.79 ± 0.18	10.42 ± 0.71
Acid, B38-CAP	0.80 ± 0.17 (0.0477*)	0.73 ± 0.22 (0.0712*)	0.87 ± 0.13 (<0.000001*)	0.73 ± 0.12 (0.0023*)	0.53 ± 0.08 (0.0002*)	3.67 ± 0.57 (0.00003*)
Acid, Spike-6P, vehicle	3.00 ± 0.45	2.93 ± 0.45	3.33 ± 0.28	3.20 ± 0.23	3.20 ± 0.37	15.67 ± 1.67
Acid, Spike-6P, B38-CAP	0.73 ± 0.19 (0.00002#)	0.87 ± 0.17 (0.0001#)	1.07 ± 0.07 (<0.000001#)	0.93 ± 0.22 (<0.000001#)	0.33 ± 0.11 (<0.000001#)	3.93 ± 0.64 (<0.000001#)

Data are shown as mean values +/- s.e.m. n = 3 for sham + Spike-6P, n = 8 for Acid + vehicle and n = 5 each for other experimental groups. One-way ANOVA with Sidak's multiple comparisons test. Numbers in parentheses show *P* values (*: Acid + vehicle versus Acid + B38-CAP and #: Acid + Spike-6P + vehicle versus Acid + Spike-6P + B38-CAP). Independent experiments were performed two times, and consistent results were obtained.

Supplementary Table 3.
Lung injury score for hamsters with RBD-Fc plus acid-induced lung injury

Group	Alveolar congestion	Hemorrhage	Neutrophil infiltrates	Alveolar wall thickness	Hyaline Membrane formation	Total scores
Control, vehicle	0.11 ± 0.07	0.33 ± 0.09	0.61 ± 0.06	0.33 ± 0.12	0.00 ± 0.00	1.39 ± 0.25
Acid, vehicle	2.54 ± 0.26	1.96 ± 0.19	2.58 ± 0.08	2.13 ± 0.15	1.96 ± 0.13	11.17 ± 0.59
Acid, B38-CAP	1.00 ± 0.18 (0.000008*)	0.92 ± 0.05 (0.00003*)	1.29 ± 0.12 (<0.000001*)	1.00 ± 0.13 (0.00001*)	0.88 ± 0.15 (0.00001*)	5.08 ± 0.44 (<0.000001*)
Acid, RBD-Fc, vehicle	3.54 ± 0.22	3.51 ± 0.20	3.71 ± 0.10	3.38 ± 0.16	3.29 ± 0.15	17.46 ± 0.67
Acid, RBD-Fc, B38-CAP	1.21 ± 0.14 (<0.000001#)	1.04 ± 0.08 (<0.000001#)	1.46 ± 0.14 (<0.000001#)	1.17 ± 0.14 (<0.000001#)	1.08 ± 0.15 (<0.000001#)	5.96 ± 0.51 (<0.000001#)

Data are shown as mean values +/- s.e.m. n = 6 hamsters for Sham + vehicle and n = 8 each for other experimental groups. One-way ANOVA with Sidak's multiple comparisons test. Numbers in parentheses show *P* values (*: Acid + vehicle versus Acid + B38-CAP and #: Acid + RBD-Fc + vehicle versus Acid + RBD-Fc + B38-CAP). Independent experiments were performed three times, and consistent results were obtained.

Supplementary Table 4. Lung injury score for hamsters infected with SARS-CoV-2

Group	Alveolar congestion	Hemorrhage	Neutrophil infiltrates	Alveolar wall thickness	Hyaline Membrane formation	Total scores
Control, vehicle	0.37 ± 0.10	0.07 ± 0.04	0.13 ± 0.07	0.23 ± 0.10	0.30 ± 0.10	1.10 ± 0.10
SARS2, vehicle	2.73 ± 0.18	2.47 ± 0.21	2.17 ± 0.14	2.37 ± 0.13	2.67 ± 0.10	12.40 ± 0.51
SARS2, B38-CAP	1.67 ± 0.23 (0.0015)	1.37 ± 0.17 (0.0004)	1.30 ± 0.22 (0.0029)	1.30 ± 0.11 (0.00002)	1.80 ± 0.18 (0.0006)	7.43 ± 0.75 (0.00001)

Data are shown as mean values +/- s.e.m. n = 6 per group. One-way ANOVA with Sidak's multiple comparisons test. Numbers in parentheses show *P* values (SARS2 + vehicle versus SARS2 + B38-CAP). Independent experiments were performed two times, and consistent results were obtained.

Supplementary Table 5. Lung injury score for hACE2 Tg mice infected with SARS-CoV-2

Group	Alveolar congestion	Hemorrhage	Neutrophil infiltrates	Alveolar wall thickness	Hyaline Membrane formation	Total scores
Control, vehicle	0.22 ± 0.11	0.00 ± 0.00	0.17 ± 0.11	0.39 ± 0.16	0.61 ± 0.10	1.39 ± 0.13
SARS2, vehicle	2.87 ± 0.23	2.40 ± 0.46	1.47 ± 0.23	2.27 ± 0.46	2.13 ± 0.27	11.13 ± 0.90
SARS2, B38-CAP	2.33 ± 0.09 (0.0427)	1.61 ± 0.10 (0.0786)	1.22 ± 0.11 (0.4781)	0.72 ± 0.23 (0.0050)	2.06 ± 0.18 (0.9532)	7.94 ± 0.29 (0.0010)

Data are shown as mean values +/- s.e.m. n = 5 for SARS2 + vehicle and n = 6 each for other experimental groups. One-way ANOVA with Sidak's multiple comparisons test. Numbers in parentheses show *P* values (SARS2 + vehicle versus SARS2 + B38-CAP). Independent experiments were performed three times, and consistent results were obtained.
