# Supplementary material for: ACE2-like carboxypeptidase B38-CAP protects from SARS-CoV-2-induced lung injury
Source: Nat Commun. 2021 Nov 23;12:6791. doi: 10.1038/s41467-021-27097-8 (PMC8610983; doi:10.1038/s41467-021-27097-8)
Supplement: Supplementary file 3 — Reporting Summary [file 41467_2021_27097_MOESM3_ESM.pdf]

nature research

Corresponding author(s): Keiji Kuba

# Reporting Summary

Nature Research wishes to improve the reproducibility of the work that we publish. This form provides structure for consistency and transparency in reporting. For further information on Nature Research policies, see Authors & Referees and the Editorial Policy Checklist.

## Statistical parameters

When statistical analyses are reported, confirm that the following items are present in the relevant location (e.g. figure legend, table legend, main text, or Methods section).

| n/a | Confirmed | |
|---|---|---|
| ☐ | ☒ | The exact sample size (*n*) for each experimental group/condition, given as a discrete number and unit of measurement |
| ☐ | ☒ | An indication of whether measurements were taken from distinct samples or whether the same sample was measured repeatedly |
| ☐ | ☒ | The statistical test(s) used AND whether they are one- or two-sided<br>*Only common tests should be described solely by name; describe more complex techniques in the Methods section.* |
| ☐ | ☒ | A description of all covariates tested |
| ☐ | ☒ | A description of any assumptions or corrections, such as tests of normality and adjustment for multiple comparisons |
| ☐ | ☒ | A full description of the statistics including central tendency (e.g. means) or other basic estimates (e.g. regression coefficient) AND variation (e.g. standard deviation) or associated estimates of uncertainty (e.g. confidence intervals) |
| ☐ | ☒ | For null hypothesis testing, the test statistic (e.g. *F*, *t*, *r*) with confidence intervals, effect sizes, degrees of freedom and *P* value noted<br>*Give P values as exact values whenever suitable.* |
| ☒ | ☐ | For Bayesian analysis, information on the choice of priors and Markov chain Monte Carlo settings |
| ☒ | ☐ | For hierarchical and complex designs, identification of the appropriate level for tests and full reporting of outcomes |
| ☒ | ☐ | Estimates of effect sizes (e.g. Cohen's *d*, Pearson's *r*), indicating how they were calculated |
| ☐ | ☒ | Clearly defined error bars<br>*State explicitly what error bars represent (e.g. SD, SE, CI)* |

*Our web collection on statistics for biologists may be useful.*

## Software and code

Policy information about availability of computer code

| | |
|---|---|
| Data collection | Bio-Rad Image lab Touch software (version 2.1.0.35), Thermal Cycler Dice Real Time System Software (version 5.11C for TP900), BD CellQuest ProTM (version 6.0), Nikon NIS-Elements (version 3.22.00), Bio-Rad Microplate Manager Software (version 6.3), FinePoint (version 2.8.0.12146), FACS Calibur (Becton Dickinson) |
| Data analysis | Bio-Rad Image lab (version 5.2), Thermal Cycler Dice Real Time System Software (version 5.11C for TP900), Excel (version 2108), Prism (version 9.2.0), BD CellQuest ProTM (version 6.0), FlowJo (version 10.8.0), FinePoint (version 2.8.0.12146) |

For manuscripts utilizing custom algorithms or software that are central to the research but not yet described in published literature, software must be made available to editors/reviewers upon request. We strongly encourage code deposition in a community repository (e.g. GitHub). See the Nature Research guidelines for submitting code & software for further information.

## Data

Policy information about availability of data

All manuscripts must include a data availability statement. This statement should provide the following information, where applicable:

- Accession codes, unique identifiers, or web links for publicly available datasets
- A list of figures that have associated raw data
- A description of any restrictions on data availability

The data that support the findings of this study are provided in the Article and its Supplementary Information. Source data are provided with this paper. GenBank accession code for SARSCoV-2, HKU-001a is MT230904.1.

# Field-specific reporting

Please select the best fit for your research. If you are not sure, read the appropriate sections before making your selection.

[x] Life sciences [ ] Behavioural & social sciences [ ] Ecological, evolutionary & environmental sciences

For a reference copy of the document with all sections, see nature.com/authors/policies/ReportingSummary-flat.pdf

# Life sciences study design

All studies must disclose on these points even when the disclosure is negative.

| | |
|---|---|
| Sample size | We performed the power analysis to predetermine sample size. |
| Data exclusions | We did not exclude the data. |
| Replication | One independent experiment was performed for Figures 1b, 2b-d, 5b, 5c and Extended Data Figures 1a-h, 3c, 3d, 6b, 6f. Independent experiments were performed two times for Figures 1c, 2f-j, 3c-l, 4b-k, 5a, 5d-h and Extended Data Figures 1i, 1j, 3a, 3b, 5a-f, 6a, 6c-e, 7a-g, 8a-f and three times for Figures 6b-g and Extended Data Figures 2a-e, 4a-h, and consistent results were obtained. |
| Randomization | The animals were allocated into experimental group in random. |
| Blinding | The investigators were blinded to group allocation during data collection and analysis. |

# Reporting for specific materials, systems and methods

### Materials & experimental systems

| n/a | Involved in the study | |
|---|---|---|
| [ ] | [x] | Unique biological materials |
| [ ] | [x] | Antibodies |
| [ ] | [x] | Eukaryotic cell lines |
| [x] | [ ] | Palaeontology |
| [ ] | [x] | Animals and other organisms |
| [x] | [ ] | Human research participants |

### Methods

| n/a | Involved in the study | |
|---|---|---|
| [x] | [ ] | ChIP-seq |
| [ ] | [x] | Flow cytometry |
| [x] | [ ] | MRI-based neuroimaging |

## Unique biological materials

Policy information about availability of materials

| | |
|---|---|
| Obtaining unique materials | We confirm that the unique materials in this study are available from us. |

## Antibodies

| | |
|---|---|
| Antibodies used | For western blotting, we used anti-mouse ACE2 antibody (Crackower, M. et al., Nature, 2002, doi:10.1038/nature00786), anti-hamster GAPDH antibody (GeneTex, GTX100118), anti-SARS-CoV/SARS-CoV-2 NP antibody (Chan, J. F. et al., Clin Infect Dis, 2020, doi:10.1093/cid/ciaa325), anti-human ACE2 antibody (R&D systems, MAB9331) , anti-beta-actin antibody (Sigma, A5316, batch |

number 123M4876) and anti-human IgG antibody (MBL, 103R, lot186).
For in vitro binding assay, we used anti-human ACE2 antibody (Novus Biological, SN0754, NBP2-67692, lotHN0420), anti-mouse ACE2 antibody (Crackower, M. et al., Nature 2002, doi:10.1038/nature00786) , anti-human IgG antibody (MBL, 103R, lot186), anti-B38-CAP polyclonal antibody (Minato, et al., Nat Commun, 2020, doi:10.1038/s41467-020-14867-z), FITC-conjugated human IgG-specific polyclonal antibody (Jackson ImmunoResearch, #109-095-088, lot137124) and Fc antibody (Jackson ImmunoResearch, # 109-035-098, lot146365).

| | |
|---|---|
| Validation | Validation information of each antibody is as follows: anti-mouse ACE2 antibody (Crackower, M. et al., Nature 2002, doi:10.1038/nature00786) , anti-hamster GAPDH antibody (https://www.genetex.com/PDF/Download?catno=GTX100118), anti-SARS-CoV/SARS-CoV-2 NP antibody ( Chan, J. F. et al., Clin Infect Dis, 2020, doi:10.1093/cid/ciaa325), anti-human ACE2 antibody (https://resources.rndsystems.com/pdfs/datasheets/mab9331.pdf?v=20211006&_ga=2.210780560.420495370.1633586814-1097249821.1598599156), anti-beta-actin antibody (https://www.sigmaaldrich.com/deepweb/assets/sigmaaldrich/product/documents/863/388/a5316blot.pdf), anti-human ACE2 antibody (https://www.novusbio.com/PDFs4/NBP2-67692.pdf), anti-human IgG antibody (https://ruo.mbl.co.jp/bio/dtl/A/?pcd=103R), anti-B38-CAP polyclonal antibody (Minato, et al., Nat Commun, 2020, doi:10.1038/s41467-020-14867-z) , FITC-conjugated human IgG-specific polyclonal antibody (https://www.jacksonimmuno.com/catalog/products/109-095-088) and Fc antibody (https://www.jacksonimmuno.com/catalog/products/109-035-098). |

# Eukaryotic cell lines

Policy information about cell lines

| | |
|---|---|
| Cell line source(s) | Vero E6 cells (CRL-1586) and Caco2 cells (HTB-37) were obtained from ATCC. Vero E6/TMPRSS2 (JCRB1819) were obtained from JCRB Cell Bank. Expi293F cells were obtained from Thermo Fisher Scientific (A14635). |
| Authentication | Vero E6 cells and Caco2 cells were authenticated with STR profiling by ATCC. VeroE6/TMPRSS2 cells was authenticated by JCRB Cell Bank, but no information of technique to authenticate is available. Expi293F cells was not authenticated. |
| Mycoplasma contamination | Not tested for Mycoplasma contamination |
| Commonly misidentified lines (See ICLAC register) | None |

# Animals and other organisms

Policy information about studies involving animals; ARRIVE guidelines recommended for reporting animal research

| | |
|---|---|
| Laboratory animals | All animal experiments conformed to the Guide for the Care and Use of Laboratory Animals, Eighth Edition, updated by the US National Research Council Committee in 2011, and approvals of the experiments were granted by the ethics review board of Akita University, NIBIOHN, the University of Tokyo or the University of Hong Kong.<br>We used 3, 6 or 10 week-old male or female Syrian hamsters and 3-4 month-old male human-ACE2 transgenic mice. Three to four month-old male or female C57BL/6J mice were used to backcross human-ACE2 transgenic mice. |
| Wild animals | No wild animals were used in this study. |
| Field-collected samples | No field collected samples were used in this study. |

# Flow Cytometry

## Plots

Confirm that:

- [x] The axis labels state the marker and fluorochrome used (e.g. CD4-FITC).
- [x] The axis scales are clearly visible. Include numbers along axes only for bottom left plot of group (a 'group' is an analysis of identical markers).
- [x] All plots are contour plots with outliers or pseudocolor plots.
- [x] A numerical value for number of cells or percentage (with statistics) is provided.

## Methodology

| | |
|---|---|
| Sample preparation | Vero E6 cells are detached by 2 mM EDTA/PBS and incubated with RBD-Fc or control-Fc protein at 4℃ or 37℃ for 3 hours. Cells were then incubated with FITC-conjugated human IgG-specific polyclonal antibody (Jackson ImmunoResearch, #109-095-088) for detection of RBD-Fc protein and control-Fc bound to Vero E6 cells. |
| Instrument | FACS Calibur (Becton Dickinson) |
| Software | FlowJo v10.8 software |
| Cell population abundance | Five thousand cells per analysis and 100% purity of live Vero E6 cells |

Gating strategy

FSC/SSC plot was gated for live and healthy Vero E6 cells.

☒ Tick this box to confirm that a figure exemplifying the gating strategy is provided in the Supplementary Information.
